# Quality improvement for parenteral nutrition in hospital: Applying a gap analysis to an electronic health record to review parenteral nutrition processing

**DOI:** 10.1002/ncp.11254

**Published:** 2024-12-18

**Authors:** Andrea Kulyk, Jolayne Dahmer, Leah Gramlich

**Affiliations:** ^1^ Faculty of Medicine & Dentistry University of Alberta Edmonton Alberta Canada; ^2^ Alberta Health Services Edmonton Zone Parenteral Nutrition Edmonton Alberta Canada; ^3^ Royal Alexandra Hospital Edmonton Alberta Canada

**Keywords:** EHR, Epic, improvement, nutrition, parenteral, quality

## Abstract

**Background:**

In light of the complex and high‐risk nature of parenteral nutrition (PN), reviewing PN processing steps is essential to minimize patient harm. The main steps include ordering, verification, compounding, and administration. Electronic health records (EHRs) have become increasingly utilized and can play a critical role in enhancing the safety of PN processin. Epic EHR is used throughout all PN processing steps within our health system. There is limited literature on health system quality improvement initiatives in PN processing.

**Methods:**

We reviewed the steps of PN processing in our health region and applied a gap analysis to assess Epic's functionality in PN processing. This gap analysis aimed to identify opportunities to enhance PN safety.

**Results:**

Epic applies 32 of 40 functions that enhance PN safety. We selected three functions to prioritize adding into future EHR iterations; these include (1) bidirectional automatic interfacing between the automated compounding device and EHR reflecting real‐time updates on product availability/shortages, (2) automatically transmitting a pharmacist‐modified PN order back to the prescriber for approval, and (3) adding additional clinical decision support tools, one of which is incorporating a 3‐in‐1 qualification calculator and the second is requiring prescriber justification for using compounded formulations over multichamber bags. Additional opportunities for improving safety in PN processing were identified and added to the gap analysis.

**Conclusion:**

Using a gap analysis is a simple process to review a health system's EHR to identify opportunities to enhance patient care.

## INTRODUCTION

Parenteral nutrition (PN) is a complex medication, containing up to 40 ingredients^1^ and requiring multiple steps in processing. The components of a PN admixture depend on the location of vascular access (peripheral or central) and the formulation chosen (premixed (comercially available multichamber bags [MCB]) vs compounded). The steps of PN processing include prescriber ordering, pharmacy reviewing/verifying, compounding, labeling, transport, and administration.^1^ Multiple healthcare professionals may be involved in the PN ordering process, including physicians, physician assistants, nurse practitioners, dietitians, and pharmacists.

The American Society for Parenteral and Enteral Nutrition (ASPEN) recommends a standardized order sequence when writing a PN prescription, including patient demographics, allergies, height/weight, vascular access device, ingredients, additives, and administration instructions.^2^ Using a standardized order sequence, PN prescriptions can be ordered and administered across various environments, including inpatient hospital wards, long‐term care centers, and patient' homes.^3^

Electronic health records (EHRs) are increasingly used to store, retrieve, and manage patient health records.^4^ Epic is among the leading EHR providers and the most adopted worldwide.^4^ Locally, we use Epic to unify patient health information across a large geographical health region to form a digitally interconnected health system by using the same software. Epic is the core technology partner (providing the user interface function)^5^ and supports computerized prescriber order entry (CPOE), pharmacy verification, label and file creation, and the electronic medication administration record (eMAR). Additional features of Epic include clinical decision support (CDS) tools and pooled data extraction for quality improvement purposes.^4^

Within our health region is a well‐developed infrastructure to optimize care for those receiving PN, the majority of which hinges on the use of Epic. The region covers an extensive geographical area and supportsapproximately two‐and‐a‐half million individuals. In light of this, nutrition and pharmacy services are centralized to provide standardized comprehensive care. There are multiple designated facilities capable of administering PN. Ground‐level nutrition care is provided by a multidisciplinary inpatient nutrition support team comprising dietitians, pharmacists, and physician nutrition specialists who review all inpatients receiving PN on a weekly basis. Registered dietitians (certified to prescribe PN by the College of Dieticians of Alberta)^6^ order the vast majority of PN within our health region. The standing Provincial PN Steering Committee, led by provincial nutrition services and pharmacy, was created in 2014 to address PN‐related health quality and guide the health system in implementing PN best practices. Lastly, “Insite,” a webpage accessible through Epic, is available to all healthcare staff and contains PN‐relevant information, including the management policy, drug shortage updates, administration/monitoring information, and the provincial PN manual, which contains detailed PN information specific to the health region.

Considering the increased uptake of EHRs across the globe, a 2015 working group comprising members of ASPEN, the Academy of Nutrition and Dietetics, and the American Society of Health‐System Pharmacists came together to identify areas of opportunity to optimize EHR use for PN workflow based on ASPEN PN Safety Consensus Recommendations.^7^, ^8^ Based on the findings from this working group, a gap analysis was developed by Ayers et al.^8^ to assess PN processing and EHR functionality.

PN is considered a “high‐alert medication,”^9^ which can cause patient harm, resulting in hospital pharmacies establishing policies to ensure safe delivery. Studies have reported high PN processing error rates; up to 20% of injectable medication errors were attributed to PN,^10^ 46% of home PN prescriptions contained at least one discrepancy,^11^ and a general error rate on reviewing PN prescriptions was 1.6%.^12^ These results argue for regular interval quality improvement assessments regarding PN processing. One such quality improvement tool is a gap analysis focusing on EHR functionality to optimize safety in PN processing. Applying a gap analysis provides a reference point for health system members to identify and address deficiencies. This information can then be used to advocate for improved processing and EHR functioning by engaging key stakeholders at a system level to drive change.

We applied a gap analysis to assess the Epic EHR's functionality for PN processing. This quality improvement process aimed to identify opportunities to enhance patient safety in PN processing within the health region.

## METHODS

This retrospective review evaluated the functionality of the Epic EHR in processing PN prescriptions for admitted hospitalized patients within our health region. The process used for this review was a gap analysis that was modified from the original Ayers publication from 2021^8^ (Table 1). The modifications to the original gap analysis reflected our goal of observing frontline users of Epic at all steps of PN processing to determine the extent to which an EHR function was implemented. The method of obtaining results for the gap analysis was done by direct observation and gathering first‐hand opinions from frontline users of Epic at the various steps of PN processes (nurses, dietitians, physicians, etc). Local ethics approval was received.

**Table 1:** Results of the modified gap analysis adapted from Ayers et al. (2021)^6^ of our electronic health record (Epic) functionality in parenteral nutrition processing.

#	Process/function	This function has not been applied	This function has been partially applied	This function has been fully applied
1	Standardized PN order forms			X
2	CPOE for PN ordering			X
3	CDS to prescribers at the time of PN order entry			X
4	CDS to pharmacists at the time of PN verification			X
5	CDS to pharmacists at the time of PN compounding			X
6	CDS to nurses at the time of PN administration	X		
7	CDS alerts for PN max total daily doses			X
8	CDS alerts for PN final concentrations			X
9	CDS alerts for PN rates of infusion that result in toxicity			X
10	CDS alerts for PN rates of infusion that result in instability			X
11	PN is included in the MAR			X
12	ACD retrieval of PN orders via scanning the PN label barcode			X
13	Direct transmission of PN orders from the EHR to ACD without manual reentry			X
**14**	**Transmission of modified PN orders back to the EHR for prescriber approval and signature** a	**X**		
15	Alerts when formulation issues are identified by the ACD			X
16	Clinical information provided by the ACD	X		
17	Integration of ACD software with existing programs		X	
18	Standardization of ACD software follows standards of ASPEN PN label formats			X
19	Standardization of additive sequence in ACD to optimize safety			X
20	Sequence of ingredients for PN admixture matches sequence in EHR, ACD, and PN label			X
21	EHR, ACD, and PN label contain matching units of the calculated total ingredients from ordered units of measure			X
22	Electronic calculation, conversion, and manipulation without manual data entry			X
***23***	Configured templates and calculations for specific patient populations		X	
**24**	**EHR and ACD interfaces simultaneously modifiable such that changes to individual product ingredients can be made to reflect availability, shortages, and/or conservation** a	**X**		
25	CDS calculation of PN component concentrations			X
26	CDS calculation of total amount of electrolytes from PN components			X
27	Barcode scanning for hanging and exchanging products used on ACDs and in compounding of PN admixtures			X
28	CDS customizable soft‐stop and hard‐stop limits for component ingredients			X
29	CDS dosing alerts with upper/lower limits for clinical effectiveness and stability (all units of measure and accounts for peripheral vs central administration)			X
30	CDS with autopopulating field that avoid manual entry			X
31	CDS with mandatory fields before order entry			X
32	Precipitation warnings for calcium and phosphorus based on appropriate calcium‐phosphate solubility curves for PN components ordered			X
33	Checkboxes and/or drop‐down menus instead of free‐text entry			X
34	Tools within EHR and ACD to capture failed message transfers	X		
35	Tools within EHR and ACD to support downtime of the interface			X
36	Outsourcing of PN compounding without manual transcription of PN order supported by EHR			X
37	Ability to order cyclic PN within the EHR			X
38	Order parameters for cyclic PN include total volume of PN to infuse and time period during which to infuse			X
39	Automatic infusion‐pump tapers and manual tapers are supported by EHR			X
40	EHR allows the provider to review, modify, and send PN orders to home‐infusion companies in a format compatible with PN ordering and labeling standards	X		
**N1**	**CDS alerts for compounding eligibility** a	**X**		
**N2**	**CDS for selecting a type of PN formulation (ie requiring prescriber to specify why compounded formulation is warranted over premixed)** a	**X**		
N3	EHR sends automated warning to review PN order no longer than every 7 days	X		
N4	All components of PN order are grouped together and easily viewable within the MAR	X		

Abbreviations: ACD, automated compounding device; CDS, clinical decision support; CPOE, computerized prescriber order entry; EHR, electronic health record; MAR, medication administration record; PN, parenteral nutrition.

^a^ Bold denotes priority function to implement.

## RESULTS

**Figure 1:**
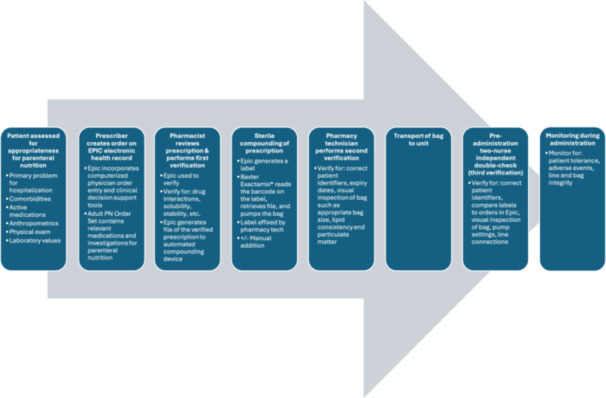
The steps of parenteral nutrition processing in our health region. PN, parenteral nutrition.

The workflow of PN processing is described as follows (Figure 1).

### Prescribing

**Figure 2:**
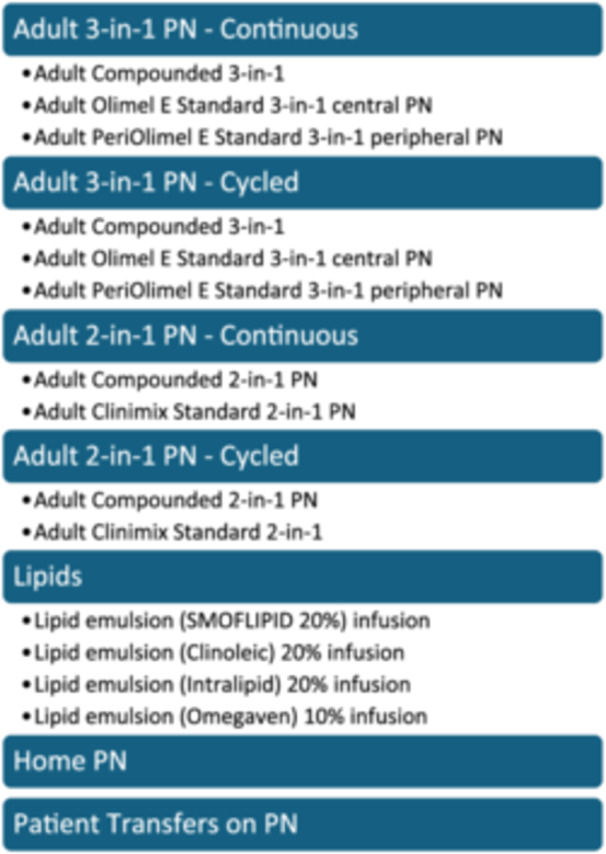
Examples and layout of the parenteral nutrition order options available within our health region as part of the Adult Parenteral Nutrition Order Set. PN, parenteral nutrition.

The order process begins with the dietitian (the usual PN prescriber in our system) using the Adult Parenteral Nutrition Order Set. Within this locally‐developed order set are one‐step links to the PN “Insite” website and the PN management policy, drop‐down orders for diet selection, glucose monitoring, and height/weight/laboratory investigations for initial/weekly monitoring, and finally, type of PN. PN formulation options are stratified by continuous vs cycled, central vs peripheral, commercially available MCBs vs compounded, and lipid injectable emulsion (ILE) infusions (Figure 2). Once a PN formulation is selected, a new order window requires input of ingredient dosages (Figure 3A). If a 2‐in‐1 order is selected, the prescriber must separately select an ILE and input the desired dose (Figure 3B). This step uses CPOE and several other safety features, including automated macronutrient percentages depending on the total dose ordered, CDS tools for compatibility, soft and hard stops, and calcium/phosphate precipitation warnings.

Figure 3(A) Example of the template a user sees when filling out an electronic order form for an adult 2‐in‐1 parenteral nutrition order. (B) Example of the template a user sees when filling out an electronic order format for an adult injectable lipid emulsion bag order. PN, parenteral nutrition.

**Figure:**
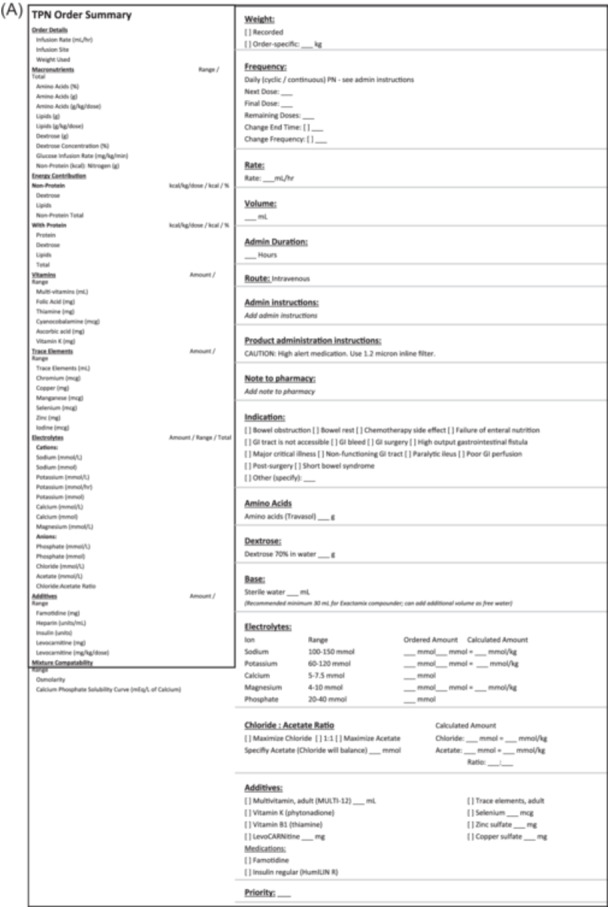

**Figure:**
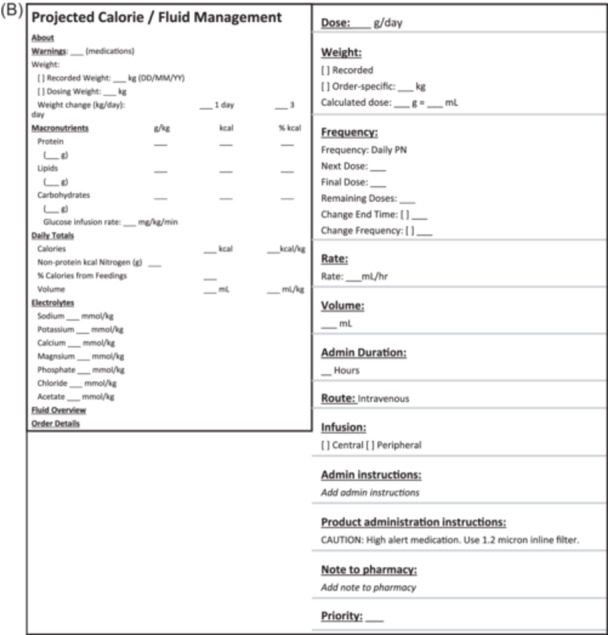

**Figure 4:**
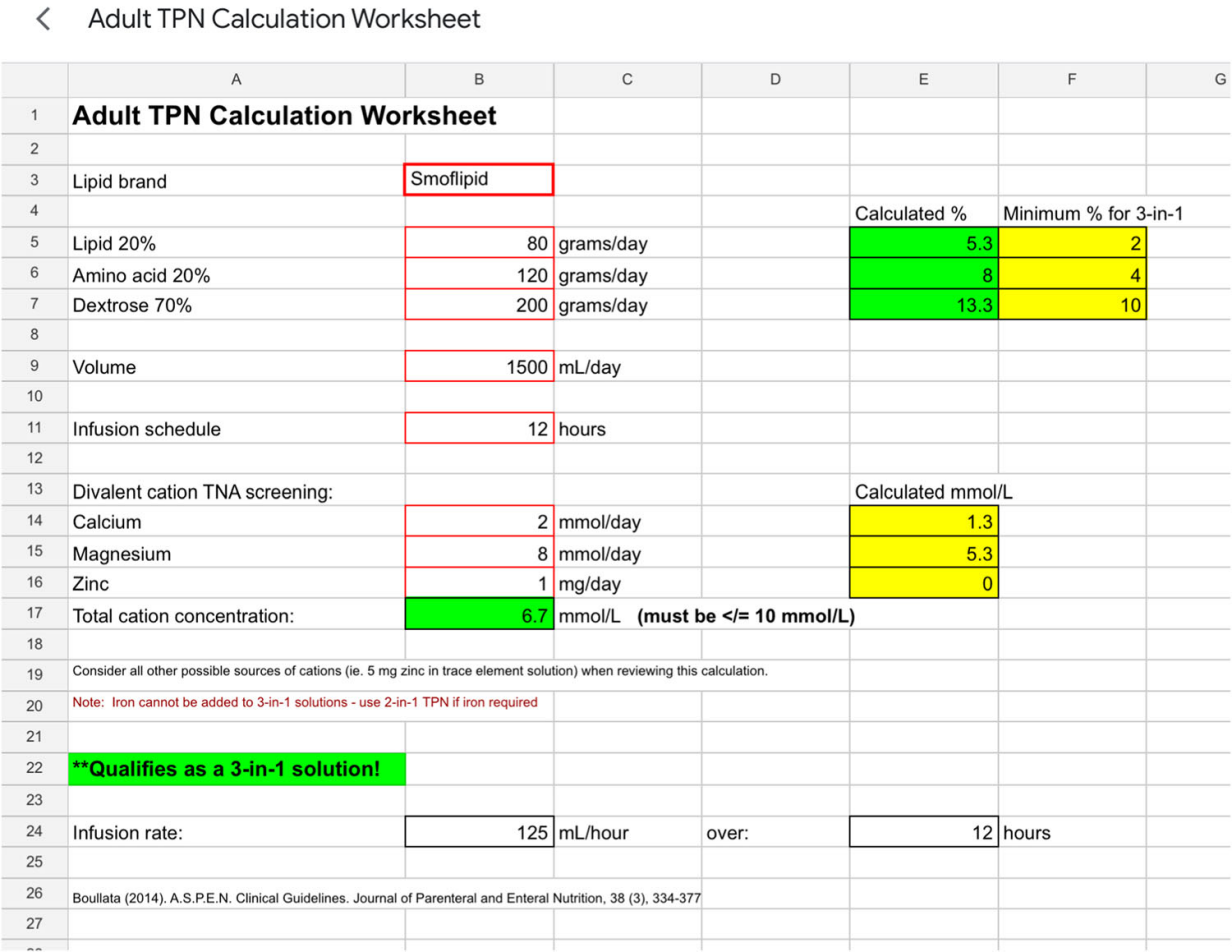
Screenshot of the parenteral nutrition 3‐in‐1 qualification electronic worksheet that includes an example of a proposed prescription that qualified for 3‐in‐1. Qualification is determined by the values input into the red‐framed cells. TNA, total nutrient admixture; TPN, total parenteral nutrition.

Producing a safe 3‐in‐1 prescription depends on meeting specific macronutrient and micronutrient quantity and total volume parameters to ensure stability. Determining whether a 3‐in‐1 prescription is safe for administration generally requires complex calculations, which can lead to errors. Dietitians use an electronic screening worksheet with formulae to calculate whether a proposed PN order qualifies (i.e., safe to administer) based on the data input into the worksheet (Figure 4).

### Order review/verification

Once an order is submitted electronically, the pharmacist receives a notification on Epic to review/verify the prescription. Pharmacist verification includes assessment for potential adverse drug reactions, toxicity, and ingredient dose/composition.^13^ If the pharmacist has a question or needs clarification regarding a prescription component, they must find the prescriber in person or send them an electronic secure chat through Epic's chat function. Similarly, should the pharmacist propose changes to a prescription, this requires approval by the prescriber (in person or via secure chat describing the proposed changes) before the prescription is sent on for compounding. The ACD does not provide real‐time updates regarding PN ingredient shortages/availability, so it is at the pharmacist verification step that temporarily unavailable ingredients must be addressed.

### Compounding

Two compounding sites within our zone compound PN bags daily for inpatients and comply with the National Association of Pharmacy Regulatory Authorities Model Standards for Pharmacy Compounding of Non‐Hazardous Sterile Preparations.^14^ These two pharmacies electronically capture all off‐site orders free of manual transcription, produce the bags, and deliver them daily. The PN order format is the same across the health region and was developed by a multidisciplinary working group with approval from the Provincial PN Steering Committee. Baxter Exactamix is the automated compounding machine (ACD) pharmacies use for compounding PN orders.

Once the pharmacist verifies a PN order, Epic generates the printed label that is manually affixed to the PN bag. The prescription is simultaneously electronically transmitted to the ACD via a digital Epic file, which automates pumping the prespecified ingredients into a bag. When additional ingredients (trace elements, multivitamins, insulin, etc.) are added, a pharmacy technician adds them under sterile conditions and uses a fixed workstation camera to photograph the specific ingredient dosage as an additional quality assurance measure.

### Final verification

A pharmacy technician uses Epic to compare the prescription order against the label on the bag (patient identifiers, ingredients, manually added ingredients with photographs, etc.) and performs a quality control assessment of the bag for bubbling or fat clumping, among other things.

### Transport and administration

The PN bags are delivered to the ward. Two nurses perform an independent double‐check to verify that the patient identifiers on the patient‐specific QR code (on their armband) match that of the PN label and that there is a corresponding PN order to administer the prescription. The administering nurse performs a second quality control assessment on the PN bag(s) for clumping and leaking. For 2‐in‐1 prescriptions, the ILE bag is hung and administered simultaneously via a Y‐port and discontinued after 12 h. The line connectivity and pump settings are checked before starting the infusion. Once the infusion has begun, the patient is monitored for tolerance and adverse effects as per nursing protocol.

The steps for reordering PN are the same, except the expiring order can be viewed in the eMAR and “reordered,” with the ability to modify the prescription as needed. Active PN orders are seen in the eMAR tab under “continuous medications” (ILEs, however, are listed under “active scheduled medications”). The steps for MCB orders are the same as those for the compounded formulations. Trace elements and vitamins are added to the MCB bag (if applicable) during the compounding step.

When transitioning patients from hospital to community, their prescriptions must be manually transcribed to a separate electronic order form and submitted directly to the home infusion company.

The results of the gap analysis are displayed in Table 1. Out of the 40 processes in the gap analysis, 32 functions are fully applied, six are not, and two are partially applied. The six functions not applied include CDS tools for nurses, the transmission of modified PN orders back to the EHR for prescriber approval, clinical information provided by the compounder, EHR and compounder interfacing simultaneously to reflect product ingredient inventory, tools to capture failed message transfers, and the ability for Epic to modify/send orders to home‐infusion companies. The two partially implemented processes are integrating compounder software into existing programs (there is only one‐way [unidirectional] communication from Epic to the ACD) and configuring templates/calculations for specific patient populations (there are templates and calculations but not for specific populations [such as neonatal, end‐stage renal, or liver disease etc.]).

## DISCUSSION

This paper reviewed Epic's functionality in PN processes by applying a modified version of a gap analysis developed by Ayers et al.^8^ Applying a gap analysis to our EHR was a simple process to initiate a grassroots quality improvement assessment in PN processing.

Completing the gap analysis revealed a significant success of Epic, which is the near‐complete elimination of manual transcription of PN orders. Compared with a 2014 survey of ASPEN and Academy members, in which only 32% had automated interfacing between EHR and pharmacy system and 28% between EHR and ACD,^15^ our EHR has automatic interfacing between the prescriber EHR to pharmacy and pharmacy to ACD. These results are significant to highlight, as Sacks et al.^12^ revealed that order transcription creates the highest number of possible errors among all the steps of PN preparation. Furthermore, Bonnabry et al.^16^ confirmed transcription is the most frequent PN error and eliminating manual transcription produced the most significant risk reduction in errors.

Some EHRs, including Epic, incorporate a CPOE that offers several benefits in prescribing PN, such as alerting for allergy–drug interactions, mandatory ordering of all PN prescription elements, and suggesting safe ranges of ingredient dosages.^17^ The increased safety CPOE offers extends beyond PN orders; Shamilyan et al. demonstrated a 66% reduction in all prescription errors after implementing a CPOE.^18^ Examples of studies assessing error rates specifically in PN after initiating a CPOE include no transcription‐associated PN errors over seven years^19^ and a reduction in prescriber errors by 61.1%.^20^

Although EHR and CPOE reduce PN errors,^1^ there is room for continued improvement in PN processing, prompting this review. Among the six EHR functions not currently applied, the functions we prioritized adding in our future EHR iterations relate to eliminating manual transcription; therefore, our highest priority is providing real‐time information on local availability, shortages, and conservations. The lack of real‐time ingredient information also had one of the lowest application rates among the hospitals included in the initial Ayers study,^8^ highlighting the universality of this gap and the focus that should be given to adding this function in future EHRs. To enable this function, future EHR iterations should include bidirectional automatic interfacing so Epic can reflect the real‐time availability of ACD ingredients. Implementing this function should reduce the number of pharmacist order clarifications, thereby reducing the number of manual adjustments to prescriptions.

The second highest priority for future EHR optimization related to PN is for pharmacists to “pend” modified PN orders in the orders section for the prescriber's final approval and signature. As described above, there is no automated transmission between the pharmacist and prescriber should the pharmacist modify a PN order. Any proposed modifications a pharmacist wants to make to a PN order require them to create a secure chat (or talk directly) with the prescriber, describe the proposed changes, and wait for a reply from the prescriber as to whether they agree or disagree with the modifications. Although Epic's strength is instantaneous communication between pharmacy and prescriber, secure chat does not allow the prescriber to view the modified prescription. Modifying PN orders relies on the pharmacist to transcribe their proposed changes, which is inefficient and prevents the prescriber from directly visualizing the impact of the PN modifications on the prescription. We suggest creating a function wherein the pharmacist sends the updated prescription back into the prescriber's orders tab as a pending order, automating an alert for the prescriber to review the modified order in a format familiar to them (ideally with the modifications being highlighted to draw attention to the changes that were made). Our prioritization of this function is based on ASPEN recommendations: “Any modifications to a PN order [should be] electronically transmitted back to the EHR system for prescriber approval and signature.”^1^ This function would enhance workflow for the approximately 5% of PN prescriptions that require pharmacist intervention.^13^

Our third priority for future EHR iterations is to add two CDS tools, one of which is to automate whether a proposed 3‐in‐1 order is safe (and therefore qualifies) for compounding (as described above), eliminating the need for a computerized worksheet (Figure 4). Currently, we follow ASPEN recommendations of using an electronic worksheet when a CPOE is not available.^1^ Although Brown et al.^21^ showed a significant reduction in PN errors by introducing a computerized PN worksheet for paper orders, it would be best to integrate this CDS feature *directly* into Epic.^22^ The second tool is to automate prioritizing MCB formulations and “push” the prescriber to select the indication as to why a compounded formula is necessary over an MCB option (eg, the patient requires more calories than an MCB can provide, severe electrolyte derangements, etc.). Our rationale for adding this tool is based on evidence highlighting the lower associated costs and similar safety profile of MCB formulations over compounded prescriptions.^22^ These two novel gaps are included in Table 1 as N1 and N2.

In addition to the prioritized two novel gaps described above, we identified two additional considerations to improve EHR functionality. It is generally recommended that an admitted patient's PN prescription be reviewed at intervals no longer than every seven days, even in patients with stable prescriptions.^1^ An EHR should “push” a notification to the most responsible physician every seven days to review the PN prescription. Secondly, in our EHR, although PN orders appear in the eMAR, ILEs are listed under the “active scheduled medications” section of the eMAR, whereas the 2‐in‐1 and 3‐in‐1 PN orders are under the “active continuous medications” section, which is confusing and cumbersome for clinicians when they are reviewing the PN prescription. All components of a PN prescription should be kept together and, ideally, a unique “PN” subheading should be included in the eMAR. These gaps were thus added to the gap analysis as N3 and N4, respectively (Table 1).

Beyond PN processing linked to Epic, our health region delivers high‐quality nutrition‐based care through diverse avenues. Across our health system, safety in PN ordering, preparation, and administration is ingrained in daily patient care. Our Provincial PN Steering Committee has incorporated several additional safety measures to administer PN safely in the hospital setting. For example, further training required by pharmacy staff includes education modules in PN‐associated EHR‐related technology. Nurses receive additional training in intravenous therapy management with vascular access devices, infusion pump management, and PN administration/monitoring. Pharmacies and nurses must both adhere to independent double‐check guidelines. Pharmacy technicians perform the final PN verification in a process commonly known as tech‐check‐tech. This format is supported by literature demonstrating pharmacist technicians to be as accurate as pharmacists in the final verification step of medication orders.^23^ Additionally, our online reporting system for patient safety events has been recently updated to improve accessibility and aid organizational learning and quality improvement. These initiatives highlight the multidisciplinary and multifaceted approach required to ensure patient safety beyond the functions identified in the gap analysis.

Dietitians play one of the most critical roles within the multidisciplinary team in ensuring the safety of PN orders. It is worth noting that dietitians almost exclusively order PN, which is a unique aspect of our health region. This practice contrasts the results of an ASPEN survey wherein dietitians were the group least often entering PN orders^3^ and among whom approximately one‐half (47%) do not have PN ordering privileges.^24^ Studies have demonstrated that involving dietitians in nutrition prescribing has improved clinical outcomes,^25^ reduced inappropriate PN prescribing,^26^ and resulted in fewer nutrition‐related order errors.^27^ Our dietitians initiated the development of a PN order set in Epic, highlighting the benefits of a multidisciplinary dietitian‐led approach to inpatient nutrition care.

Although our gap analysis focused on hospital EHR function, a key component of hospital care is ensuring a safe transition upon discharge, which is reflected in gap‐analysis function 40 (Table 1). The transition of PN prescribing from hospital to home environment is challenging globally^7^ and locally. The ability to transmit home PN orders via Epic would eliminate manual transcription, reduce potential errors, and seamlessly transition prescriptions from the hospital to outpatient environments. Unfortunately, this feature is not locally feasible for us because of third‐party involvement. However, our home PN order forms adhere to ASPEN standards^28^ and contain the same concentrations, dose units, and ingredient lists to minimize transcription‐related errors.

Most recent data suggests that approximately 86% of ASPEN members use EHRs,^3^ highlighting their essential role in PN processing. This same study also demonstrated a positive correlation between affirmative responses between the safety/effectiveness of their EHR regarding writing nutrition orders and the duration since EHR installation.^3^ As familiarity with EHRs grows, comfort with its format will likely increase. These results should reassure institutions considering transitioning from paper to EHR PN processing. As described in the Results section, our PN order set was developed and customized to reflect locally available PN bag options. Other health systems can use Figures 2, 3A, B as examples of PN order set formats.

Beyond the gaps identified in our Epic described above, our review included several weaknesses. The main weakness was the lack of a quantitative audit of PN processing to go along with the qualitative analysis of EHR functionality. The intent of our work was not to perform an audit of PN processing before and after implementing the use of Epic, because multiple studies have already shown reduced error rates when transitioning from paper to EHR‐based PN processing^12^, ^19^, ^20^ This being said, future studies should incorporate descriptive and quantitative quality improvement components (examples include surveys, focus groups, and quantification of PN‐associated errors before and after EHR updates) to enhance the validity of their findings. Another weakness is that our results are based on our regional version of Epic, which may or may not be translatable to other regions or EHRs.

## CONCLUSION

PN is a highly complex medication and has multiple steps in its processing. The risk of errors is high, with significant consequences when mistakes occur. It is crucial to regularly review and enhance safety features to minimize patient harm. Our EHR, Epic, applies the majority of functions recommended for optimizing patient safety relating to PN but still has ongoing functional gaps, as evidenced by the gap analysis. Applying a gap analysis was a simple quality improvement approach that assisted in identifying specific gaps in EHR functionality and highlighted the overall successes of our EHR in ensuring safe PN provision. The gap analysis is, however, not all‐encompassing and requires a thoughtful approach to identify potential additional gaps. Each EHR is unique, reflecting its adaptation to the health region's needs; therefore, frontline users with experience using these functions are in the best position to assist in identifying gaps in EHR functionality. Other health regions can use our Epic‐based PN workflow summary to reflect on their local PN processes. Future studies looking to enhance PN safety can consider assessing EHR functionality and incorporating both a gap analysis and an audit to evaluate for quantifiable improvement in PN‐associated error rates.

## AUTHOR CONTRIBUTIONS

Andrea Kulyk, Jolayne Dahmer, and Leah Gramlich contributed to conceptualization; Andrea Kulyk contributed to drafting and visualization; Andrea Kulyk and Leah Gramlich contributed to editing; Jolayne Dahmer and Leah Gramlich contributed to provision of resources; Jolayne Dahmer contributed to reviewing; and Leah Gramlich contributed to provision of resources supervision.

## CONFLICT OF INTEREST STATEMENT

None declared.
